# Effects of a Graphene Heating Device on Fatigue Recovery of Biceps Brachii

**DOI:** 10.3390/bioengineering10030381

**Published:** 2023-03-21

**Authors:** Wenming Liu, Xiaohui Jiang, Zhiran Yu, Kai Pang, Jian Wang, Yuxin Peng

**Affiliations:** 1 Department of Sports Science, Zhejiang University, Hangzhou 310058, China; 2 The MOF Key Laboratory of Macromolecular Synthesis and Functionalization, Department of Polymer Science and Engineering, Zhejiang University, Hangzhou 310027, China

**Keywords:** fatigue recovery, far-infrared, graphene film, heating device

## Abstract

Far-infrared (FIR) is considered to be an ideal method to promote fatigue recovery due to its high permeability and strong radiation. In this paper, we report a flexible and wearable graphene heating device to help fatigue recovery of human exercise by using its high FIR divergence property. This study compares two different fatigue recovery methods, graphene far-infrared heating device hot application and natural recovery, over a 20 min recovery time among the male colleges’ exhaustion exercise. Experimental results show that the achieved graphene device holds excellent electro-thermal radiation conversion efficiency of 70% and normal total emissivity of 89%. Moreover, the graphene FIR therapy in our work is more energy-efficient, easy to use, and wearable than traditional fatigue recovery methods. Such an anti-fatigue strategy offers new opportunities for enlarging potential applications of graphene film in body science, athletic training recovery, and wearable devices.

## 1. Introduction

Since the training load and competition frequency of elite athletes are steadily increasing and performance differences between race winners and losers are becoming more and more marginal [1], athletes have to face the difficult task of maximizing training load and adaptation whilst avoiding insufficient recovery, which would lead to maladaptation, reduced performance, and increased risk of injury [2]. Rapidly increasing strength of competitive sports and developing national fitness have posed significant challenges to fatigue recovery measures [3]. Hence, efficient fatigue recovery is becoming a critical issue for improving the performance of athletes and relieving fatigue diseases. In response to these needs, a variety of treatment modalities (e.g., massage, relaxation activities, hydrotherapy, cryotherapy, electrical stimulation, cold-water immersion, hot–cold contrast baths, low-level laser, and stretching) [4,5,6,7] have been used as the main methods to enhance recovery during rest intervals in matches or between training sessions [8].

However, many conventional treatment modalities still usually suffer from low recovery efficiency, limited conditions for use, and lack of wearability. In recent years, numerous studies have reported that nanomaterials can not only be used as flexible, stretchable, and wearable sensors [9,10], but can also become an important method for fatigue recovery, especially far-infrared (FIR) emissive materials. Far-infrared radiation, an invisible electromagnetic wave with a characteristic wavelength of 5.6 to 1000 μm [1], is known for its radiative cooling function. It can be perceived as heat by the skin and has the ability to reflect heat energy from the sun, making it highly penetrating and radiative [11,12]. Therefore, it holds great potential for fatigue recovery of human muscles. Athletes may utilize various FIR techniques, such as FIR cabins/saunas, FIR ray devices, and the latest FIR-emitting garments. However, the requirement for precise molecular structures of current far-infrared ceramics and the laborious production process significantly reduces the product yield rate and manufacturing efficiency. In addition, the lack of wearability of far-infrared ceramics is also a formidable obstacle.

To achieve advanced far-infrared emissive materials with optimal performance, it is necessary to employ scalable and cost-effective fabrication approaches that incorporate novel chemicals, synthetic routes, and methodology. Graphene, an atomically thin sheet of carbon atoms arranged in a 2D hexagonal lattice [13], has attracted numerous attention for advanced materials due to its unique thermal and electrical conductivity, high mechanical strength, and optical properties [14,15,16]. It offers great potential for next-generation far-infrared emissive materials [17]. Macroscopic graphene film assembled from graphene sheets is expected to be an ideal far-infrared emissive material for physical therapy devices due to its combined merits of high far-infrared emissivity and flexibility [18]. However, there is no study on the effects of graphene on muscle recovery after a fatiguing activity based on its far-infrared divergence properties.

Fatigue may impair the joints’ kinesthetic and proprioceptive properties [19]. It increases muscle spindle discharge threshold, which interrupts afferent feedback, consequently altering joint awareness [19] affecting static postural control [20], and to a lesser degree, dynamic postural control [21,22,23,24,25]. At present, many studies have evaluated different methods for fatigue recovery [26,27]. However, the current research on FIR in the field of sports is mainly aimed at the whole body, and little research has been carried out on local fatigue recovery. In the field of competitive sports, the biceps brachii is the most important muscle in human upper limb movement, such as throwing motion, swing motion, lifting motion, and shooting motion. Muscle fatigue will affect the joint stability and athletic performance of athletes. It is of great significance to study the recovery effect of FIR on local fatigue of biceps brachii, so the effect of graphene heating film on fatigue recovery of biceps brachii is explored in this paper.

## 2. Materials and Methods

### 2.1. Materials

The graphene-based heating device (product number BZA170) used in this study was made by Hangzhou Gaoxi Technology Co., Ltd. (Gaoxi Technology, Hangzhou, China), which has almost the same far-infrared emission spectrum as the graphene heating product (No. HYY-118B) prepared by Grahope New Materials Technologies Inc. (GNM, Shenzhen, China) [18,28], and the former heating film is much more flexible and thus more suitable for this study than the latter. The graphene heating film was made by a continuous blade coating method with an aqueous slurry of graphene and additives. The film was cut into 8 cm × 15 cm piece, connected with two electrodes of copper foil, and covered with an insulation and flame retardant fabric, subsequently connected by a temperature controller and a power adaptor, affording the graphene heating device. As the device was turned on, electric energy was converted into heat energy and radiated out in the form of far-infrared wave. The rated power of the device was settled at 5 W, and the operating temperature was adjusted by the temperature controller between 30 °C and 55 °C.

### 2.2. Preparation

**Figure 1:**
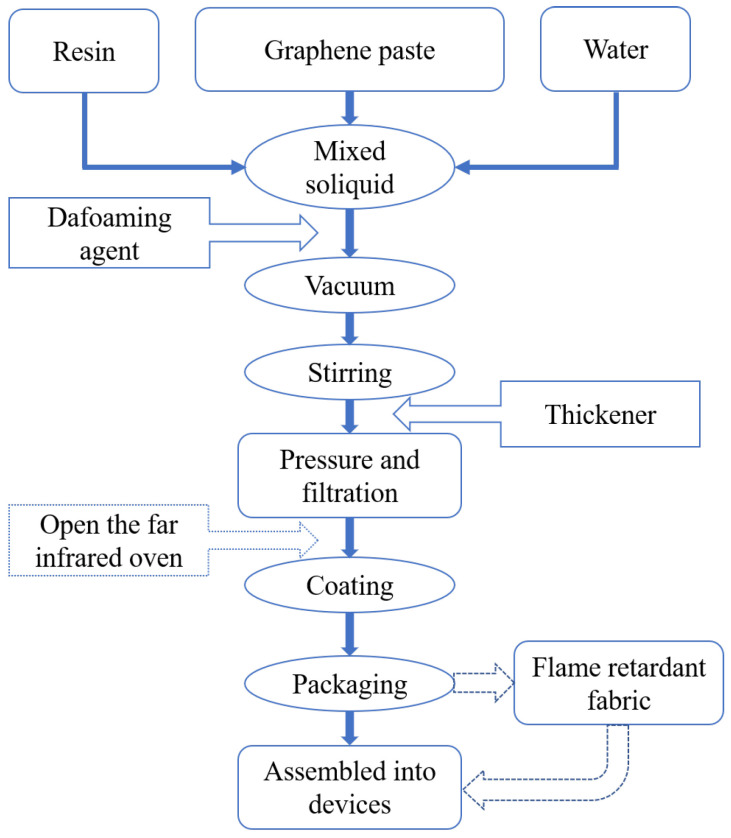
Flow chart of the graphene heating device prepared by the FFGS method.

Here, we manufactured voltage-power adjustable flexible graphene heating devices, which have been utilized as heating elements in commercial body care products (G-Phoenix) (Gaoxi Technology, Hangzhou, China). Figure 1 illustrates the fabrication process of the flexible graphene heating devices by the film-forming of graphene slurry (FFGS) method. The FFGS technique is described as follows. The graphene paste and resin were mixed with deionized water. A dispersant was then added to the mixture to form a stable soliquid. After the stirring procedure, the soliquid became highly dispersed with low viscosity. The soliquid was further evacuated and defoamed. After that, the defoaming agent and the thickener were added; the soliquid was pressurized and filtered to coat into a film under the irradiation of a far-infrared oven. Finally, the graphene heating device was assembled from the graphene film using flame retardant fabric, power, transformer, etc.

**Figure 2:**
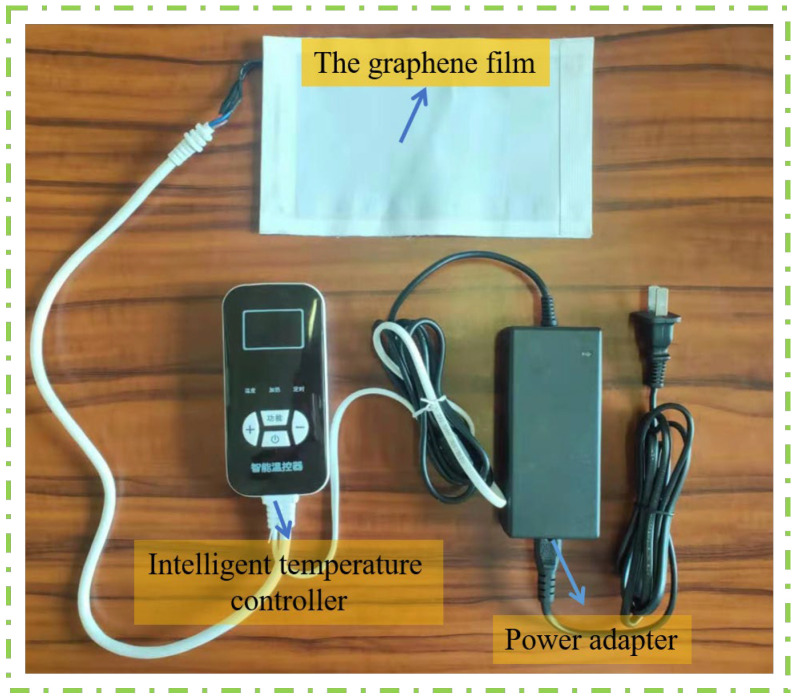
Physical picture of the graphene heating device.

Using the FFGS technique, we successfully fabricated a novel graphene far-infrared transmitter device with adjustable dimensions (Figure 2). The graphene heating device consists of three parts: the graphene film, the intelligent temperature controller, and the power adopter. It offers several advantages for infrared healthcare products: (1) it allows direct contact with the skin and can be heated automatically with adjustable temperature and voltage; (2) it has high intensity and abrasion resistance; (3) it can be prepared into different shapes to fit different parts of the human body; and (4) it exhibits high far-infrared emission efficiency and provides a comfortable warmth sensation.

### 2.3. Study Design

This pilot study was a prospective, randomized, controlled clinical trial. The recruited subjects who came to the lab for the study signed an informed consent form. This study was approved by the University Institutional Review Board and all participants provided written informed consent.

### 2.4. Participants

Thirty-six college students (all males) from general physical examination normal subjects of Zhejiang university were recruited to participate in the study. Thes inclusion criteria were: (1) healthy men; (2) age between 18–22 years old; (3) body mass index < 24 kg/m^2^; (4) right-handed. The exclusion criteria included: (1) previous injuries in the upper limb; (2) history of upper limb surgery; (3) cardio-respiratory problems; (4) history of neuromuscular or neurological pathologies; (5) inability to comply with fatigue protocol; (6) lack of consent. All participants were instructed to refrain from heavy exercise 48 h before testing to minimize confounding effects of physical activity levels. Table 1 illustrates the subject characteristics of groups A, B, C, and D. No significant difference was recorded between the groups in age, height, weight, and body mass index (*p* > 0.05).

### 2.5. Procedures

**Figure 3:**
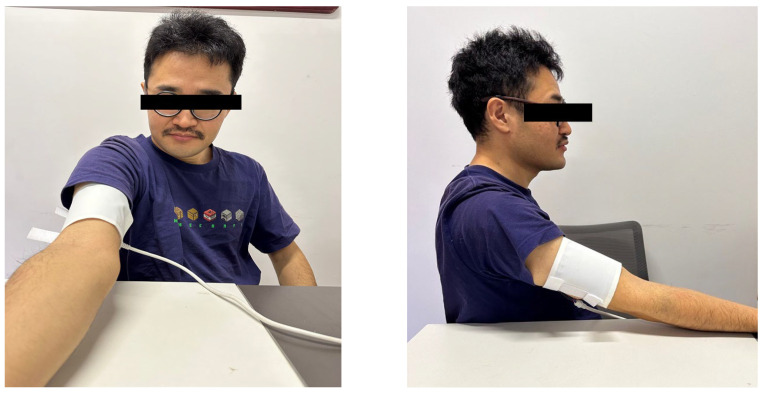
Wearing diagram of the FIR device.

After taking a medical history and completing a physical examination, the participants’ age, body mass index (BMI, calculated as weight/height^2^ [kg/m^2^]), shoeless height (cm), and shoeless weight (kg) were determined, and the informed consent forms were signed. The participants were then randomly assigned into 4 groups: (A) control group, which received no graphene device treatment; (B) the 35 °C experimental group, which received graphene device treatment with 35 °C; (C) the 40 °C experimental group, which received graphene device treatment with 40 °C; and (D) the 45 °C experimental group, which received graphene device treatment with 45 °C. All tests and fatigue rating scale (RPE) showed as follows (Table 2) were conducted to evaluate the subjects at baseline before inducing muscle fatigue (T1), immediately after completing the fatigue protocol (T2), and after treatment for 20 min (T3). The measurements were made by a physiotherapist who was aware of the subjects’ group assignments. The FIR device is worn as in Figure 3. The FIR device was attached to the surface of the biceps brachii, and the heating end was placed on the upper end of the biceps brachii.

### 2.6. Measurement

- Isometric peak force measurement

Measures of peak torque (PT) of the bicipital muscle of the arm were obtained on a Biodex System 4 Pro isokinetic dynamometer (Biodex Medical Systems, Inc., Shirley, New York, NY, USA) [29]. According to the manufacturer’s recommendations, calibrations were performed before the tests, and the axis of the dynamometer was aligned with that of the glenohumeral joint. Gravity corrections were employed during the tests. Conforming to the literature, all movements were carried out in the concentric-concentric mode [30,31]. All participants were given an explanation about the testing equipment and the procedure of assessment. The participant was tested in a sitting position with the chest and both shoulders against the chair seat, and the hip and knee angles at 90° [32]. The participants’ trunk was stabilized by two crossed straps from the contralateral shoulder across the chest and fixed with a buckle. Another strap was secured to the quadriceps table to fix the pelvis, and this position was stabilized by the other hand gripping a handle on the side of the Biodex chair. The ROM was from 0° to 120°. To ensure a high degree of subject compliance, our measurements were repeated if the coefficient of variance between the three repetitions was higher than 10%. One attempt with 3 repetitions was fulfilled in submaximal contractions (enough effort to clearly feel the resistance) in the same circumstances as the real tests. This trial was purposed to familiarize and prepare the participants for the real maximal test.

- Fatigue protocol

The sitting posture dumbbell arm bend test (SDT) was used to induce biceps muscle fatigue. Fatigue was defined as a reduction in the force production of >30% of the peak force compared to the baseline pre-fatigue value. Exercises had been started at 70% of the three-repetitions maximum load of the individual previously determined, which consisted of 8–12 repetitions at above four sets at each session [33]. Between repetition sets, resting periods were 30 s approximately [34]. The participants were verbally encouraged to complete more sets until they could no longer perform the standard action.

- Treatment

Group B, group C, and group D received graphene heating membrane therapy after exercise. After the preliminary experiment, the hot compress time for biceps muscle fatigue was determined at 20 min for the best effect. The hot compress temperatures were set at 35 °C, 40 °C, and 45 °C, respectively, to compare and analyze the effects of different temperature settings of graphene heating film on the fatigue recovery of biceps brachii. Serving as the control group, group A received the same period of static recovery for 20 min.

### 2.7. Statistical Analysis

The initial torque difference between different groups was tested by an independent sample t-test. A control group and 3 experimental groups (with temperature: 35 °C, 40 °C, and 45 °C) mixed factor analysis of variance (ANOVA) was conducted on muscle strength loss and recovery. Separate three-way mixed factor ANOVA was conducted to test the main interaction effects with and without graphene heating device, different temperatures (35 °C, 40 °C, and 45 °C) and phases (before experiment, after experiment, and after recovery of 20 min) on peak torque and stimulus for biceps brachii. The level of significance for all analyses was set at *p* < 0.05. Where required, post hoc comparisons were performed using simple effects tests with a predetermined alpha of 0.05. All analyses were conducted using SPSS 26 (IBM SPSS Statistics, New York, NY, USA).

## 3. Results

Graphene, a two-dimensional (2D) material, possesses remarkable mechanical strength (130 GPa), exceptional thermal conductivity (5300 W/mK), and superior electrical conductivity (108 S/m) [35,36]. Additionally, graphene exhibits excellent far-infrared radiation properties [18] and has a unique electronic structure that leads to distinctive optical properties [37]. It emits far-infrared light in the range of 8~15 μm, which coincides with the human body scattering band [18]. This characteristic makes it easily absorbed by human tissues, inducing a resonance effect that can penetrate far-infrared heat into subcutaneous tissues, promote blood circulation, dilate capillaries, and accelerate metabolism. Numerous studies on graphene have shown its unique properties in various research fields, which have generated new possibilities for the exploration of potential applications.

Far-infrared ray with strong penetration force and radiation force is easy to absorb by the object and turn into its internal energy [38]. It has a significant temperature control effect and resonance effect. When it is absorbed by the body, the blood flow in the skin is increased, the exchange between blood and tissue will be accelerated, and the toxin will be expelled from the body faster [39]. While the human body is made up of about 70 percent water, the far-infrared ray improves recovery efficiency by enhancing water activity. When infrared radiation penetrates through water, water molecules will absorb the radiant energy and the intermolecular forces will be reduced [40]. Large molecule groups will become smaller molecule groups. Therefore, the water density will be increased and water activity will be improved. Under the influence of infrared radiation, the effect of the penetration, proliferation, dissolution, cleaning, and emulsification of water will be enhanced and the metabolism will be accelerated. Recently, infrared radiation has been successfully used for healthcare [41], for the treatment of arthralgia, curvature, traumatic injuries, and the prolapse of the lumbar intervertebral disk and bone injuries.

Here, the graphene heating device was directly pasted on the fatigue site for the study of fatigue recovery. The specific experimental results are as follows. The peak torque, average peak torque, average peak torque per unit weight, coefficient of variance, and stimulus before/after exercise and after treatment within each group are shown in Table 3. There is neither a significant difference recorded between groups before and after exercise in peak torque, average peak torque, average peak torque per unit weight, coefficient of variance, and stimulus, nor significant difference recorded in group A before and after treatment in these data (*p* > 0.05). Nevertheless, there were significant increases before treatment compared with that after treatment in groups B, group C, and group D. Particularly, the peak torque, average peak torque, average peak torque per unit weight, and stimulus of group C after treatment were significantly different from those before treatment (*p* < 0.001). Taking the average peak torque as the standard, the fatigue rates of group A, group B, group C, and group D were, respectively, 66.76%, 69.01%, 69.18%, and 68.01% as calculated by the following formula. These dates were all less than 70% and there was no significant difference between each other. Similarly, the recovery rates of these four groups calculated by the formula below were 74.64%, 89.14%, 88.01%, and 84.72%, respectively. The difference between fatigue rate and recovery was 7.88%, 20.13%, 18.83%, and 16.71% among these four groups, which means that the recovery rate in the experimental groups treated with the graphene heating device was two~three times that of the normal control group.

The rate of fatigue = (after exercise)/(before exercise) × 100%

The rate of recovery = (after treatment)/(before exercise) × 100%

Difference = the rate of recovery − the rate of fatigue

The rank of immediate subjective feelings after exercise and post-treatment is shown in Table 4. There was no significant difference recorded between groups after exercise on the self-perception scale (*p* > 0.05). However, there were significant increases between groups post-treatment on the self-perception scale (*p* < 0.001). In addition, compared with group A, there were significant increases in group B and group C post-treatment on the self-perception scale.

## 4. Within-Group Comparison

Average peak torque was significantly increased after treatment compared with that before treatment in groups C (*p* < 0.001). In addition, a significant increase in group B (*p* < 0.01), and a little increase in group D (*p* < 0.05) were found, while no significant difference was found in average peak torque in group A (*p* > 0.05). (Table 5).

Fatigue level was significantly increased after treatment compared with that before treatment in groups A, B, C, and D (*p* < 0.001) (Table 5).

## 5. Between-Group Comparison

No significant difference was recorded between groups in average peak torque and fatigue level before treatment (*p* > 0.05).

No significant difference was noted in group A compared with the mean values of group B, group C, and group D after treatment (*p* > 0.05). Moreover, there was no significant difference recorded in the average peak torque of group B compared with that of group D after treatment and in the average peak torque of group C compared with that of groups B and D after treatment (*p* > 0.05) (Table 6).

A significant increase in average peak torque was reported in group A compared with the mean values of group C and group D after treatment (*p* < 0.05), while no significant difference was noted in group A compared with the mean values of group B after treatment (*p* > 0.05) (Table 6). Moreover, there was no significant difference recorded in the average peak torque of group B compared with that of group D after treatment and in the average peak torque of group C compared with that of group B and D post-treatment (*p* > 0.05).

## 6. Discussion

The aim of the current study is to verify the effects of different graphene heating film temperatures on the fatigue recovery of biceps brachii. Muscle fatigue can be defined as an exercise-induced decrease in force-generating capacity during maximal voluntary muscle contractions (MVC), or the inability to further sustain an exercise at a required force [42,43]. We hypothesized that the higher the temperature, the better the recovery effect of biceps brachii. According to the experimental results, compared with the control group, the graphene heating film at three different temperatures can promote fatigue recovery. In particular, the peak torque, average peak torque, average peak torque per unit weight, and stimulation difference at 40 °C recovery are very significant. In addition, the self-perception scale increased significantly after treatment at 35 °C and 40 °C.

Generally speaking, it is difficult for athletes to fully recover their muscles in a short time to reach the best competitive state. The effect of physical therapy such as cold-water bath on fatigue recovery has been studied. Likewise, muscle fatigue has been assessed through isometric force fall in response to MIVCs performed before and after different exercise protocols [44,45,46]. In the present study, the isometric exercise bout was able to induce a fatigue condition, as evidenced by the reduced isometric strength immediately after exercise (Table 4). Previous literature has studied various cooling methods (such as ice bag, CWI, and ice massage) to reduce skin temperature and promote fatigue recovery [47]. Although it was proved that CWI had a good response to muscle soreness, no dose–response relationship was observed for any outcome based on the application of the temperatures used [45], such as countermovement jumps, squat jumps, drop jumps with sledge apparatus, and sprints, but the experimental results did not find any benefit from using cold-water immersion compared to other recovery interventions [48]. Delextrat et al. compared the effects of intermittent cold-water immersion and massage on perceptual and performance markers of recovery by basketball players after competitive matches. The results found that massage is less useful than cold-water immersion in recovery [49]. There are also studies that combined multiple recovery interventions. A study compared five recovery methods and the results showed that, for short-term perceptual recovery, contrast water therapy (CWT) should be implemented and for short-term countermovement, power performance and active or control recovery is desirable [50]. Table 7 shows a comparative analysis of the effectiveness of various methods of fatigue recovery, including the research findings of this study. The low recovery efficiency and limited applicability of traditional recovery methods have led scholars to consider nanomaterials as a crucial avenue for research on fatigue recovery. Katsuura et al. and Nunes et al. found that wearing bioceramic clothing had no significant effect on fatigue recovery [51,52]. HE et al. compared massage therapy, ordinary far-infrared therapeutic apparatus, and far-infrared ceramic bead therapy [53]. The results showed that the recovery effect of ceramic beads was much better than the other two groups. Meanwhile, Soejima et al. suggest that WA on therapy employing an FIR dry sauna may be useful for treating chronic fatigue syndrome [54]. However, the application of WA in therapy has some limitations due to the harsh conditions of use. Materials with high far-infrared emissivity are quite promising in the modern medical care field, owing to their accelerating effects on blood circulation and metabolism [55]. For example, the far-infrared ceramic, invented by Professor Yang of Tsinghua University, has a significant infrared emission function, and the far-infrared emission rate of 2~18 μm at 60 °C is 88~92%, which is widely used in the training and recovery of professional athletes and the daily healthcare physiotherapy of ordinary people [38]. However, due to the complexity and low efficiency of the production process of far-infrared ceramics, it is difficult to apply it to the fatigue recovery of sports. Therefore, we employed far-infrared graphene material to explore its effect on the fatigue recovery of biceps brachii. Our study proved that the graphene FIR device could significantly alleviate fatigue after one-time exhaustion, and the degree of self-perceived fatigue was also significantly reduced, but other research reports have no significant changes [56]. Therefore, the results in this study revealed that we can promote fatigue recovery through the thermal effect and resonance effect of the FIR graphene heating device.

FIR has three main biological effects: radiation, vibration (or resonance), and thermal effect [58]. Radiation and resonance promote the oscillation of free ions, resulting in the denaturation of macromolecules such as proteins leading to an increase in the absorption of proteins in tissue frameworks [59]. Thermal effects induce angiogenesis and promote microcirculation by expanding blood and lymphatic vessels, activating Langerhans cells and macrophages [60]. At the same time, the Arginine-Modified Carbon Surface has great application in the development of electrochemical sensing, especially in the detection of dopamine [61]. The far-infrared graphene heating film can emit 8~15 μm infrared light, and the infrared emissivity is as high as 89%, which indicates a healthy infrared material. The biological effects of far-infrared rays have two aspects: far-infrared thermal biological effect and non-thermal biological effect. Compared with other healthcare materials, graphene films with these two kinds of biological effects on fatigue recovery have a positive effect. They have a good thermal effect and resonance effect, making the biomolecule at a higher resonance level, improving microcirculation, and promoting blood circulation and metabolism. When the infrared power is absorbed by the human body, it causes vibration in the myofibril molecule peptidoglycan, and in the case of low ATP, the biological compensation is transferred smoothly from one place to another. One study has proved that the temperature change caused by far-infrared radiation of graphene can be used to judge the strength of the human body’s “Yang Qi” [62]. Furthermore, graphene thermal therapy can reduce the maximum diameter and maximum cross-sectional area of benign thyroid nodules, which has a good clinical application prospect [63]. These effects suggest that the far-infrared graphene heating film has a good effect on fatigue relief, though its mechanism in exercise fatigue intervention recovery is still unclear.

**Figure 4:**
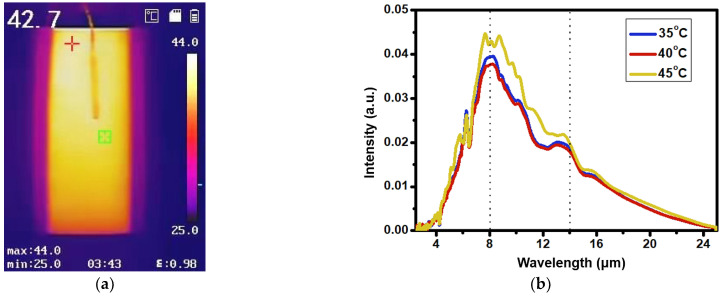
(**a**) Infrared thermal image of graphene heating at 42 °C; (**b**) Far-infrared emission spectra of graphene heating device at different temperatures.

Figure 4a shows the infrared thermal image obtained by the graphene heating device using the infrared thermal imaging instrument at 42 °C. The temperature scale indicates that the white color represents the material’s strongest infrared radiation, while the black color represents its weakest infrared radiation. We can see from the figure that the radiation of the graphene heating device is uniform, and the emissivity of the graphene heating film is 0.98, which is much higher than other materials (the galvanized glass is 0.05, the copper is 0.57, the sea salt is 0.91, and the far-infrared ceramic beads is 0.94) [40]. Meanwhile, there is no significant difference in the far-infrared spectra of the graphene heating device at different temperatures of 35 °C, 40 °C and 45 °C; only the intensity is different at 45 °C, as shown in Figure 4b. This indicates that the heating temperature has no obvious effect on the wavelength and intensity of the far infrared. Furthermore, the far-infrared emission material exhibits a consistent infrared wavelength range at three distinct temperatures, making it well-matched with blackbody radiation spectra. With an appropriate temperature, optimal thermal comfort can be maintained by the graphene heating device, which makes it an effective wearable device for promoting fatigue recovery.

The current study evaluated the impact of adding far-infrared hot compress supplementation to an exercise training program on 36 patients with biceps fatigue, who were randomized into four equal groups. Results showed that peak torque was significantly increased in groups B, C, and D post-treatment compared with the mean values of pre-treatment. In addition, a significant increase was recorded in the peak torque of group C compared with that of group A post-treatment. Compared with other traditional fatigue recovery methods, even other far-infrared technology, the graphene far-infrared device has a better effect on human local fatigue recovery. It is easier to carry and use, which is more conducive to application in competitive sports. In addition, the graphene heating device in this paper is more flexible when it has the same emission spectrum as other products, so it is more suitable for fatigue recovery in sports [18]. Therefore, this work proves that the graphene heating device has a promising application for human local fatigue recovery.

## 7. Conclusions

In this work, we report the preparation of a graphene far-infrared heating device and compare the effects of its intervention on the fatigue recovery of male college students after exhaustion. Compared with the natural recovery approach, a positive effect was found in terms of self-reported sensory manifestations in fatigue, as well as on the subjects’ maximum torque peak, suggesting that the graphene far-infrared heating device contributes to the recovery of the biceps muscle from exhaustion fatigue. In the future, we will upgrade our device to be rechargeable and portable, so that it can be applied to multiple conditions. In addition, we will apply the device to the muscles of the lower extremities to include the whole-body motion, and thus to sports that are not limited to the use of the upper limbs.

## Figures and Tables

**Table 1:** General characteristics and grouping of participants.

	Group A	Group B	Group C	Group D
	Control Group	35 °C	40 °C	45 °C
	Mean ± SD	Mean ± SD	Mean ± SD	Mean ± SD
n	9	9	9	9
Age (years)	19.44 ± 1.13	19.55 ± 1.33	19.13 ± 1.13	20.11 ± 1.36
Weight (kg)	71.72 ± 4.81	71.72 ± 5.82	70.13 ± 6.24	67.82 ± 3.71
Height (cm)	178.44 ± 5.20	180.7 ± 6.93	177.75 ± 5.75	177.67 ± 4.06
BMI (kg/m^2^)	22.53 ± 1.37	21.95 ± 1.31	22.13 ± 1.20	21.50 ± 1.31

The Group 35 °C, 40 °C and 45 °C means treatment with graphene device at temperature of 35 °C, 40 °C and 45 °C.

**Table 2:** Fatigue rating scale (RPE).

Self-Perception	Grade
No sweat at all	6–7
Very easily	8
Easily	9–11
Easy	12–13
A little tired	14–15
Tired	16–17
Very tired	18
Quite tired	19
Burnout	20

**Table 3:** Four index contrast of peak torque (Nm), average peak torque (Nm), etc., before exercise, after exercise/before treatment, and after treatment.

	Peak Torque (Nm)	Average Peak Torque (Nm)	Average Peak Torque per Unit Weight (%)	Coefficient of Variance (%)	Stimulus (Nm)
	Mean ± SD	Mean ± SD	Mean ± SD	Mean ± SD	Mean ± SD
Before exercise					
A	57.70 ± 9.57	55.17 ± 9.27	77.56 ± 14.89	4.52 ± 2.26	11.02 ± 1.86
B	53.80 ± 6.60	51.48 ± 6.17	72.04 ± 7.02	4.38 ± 3.01	10.30 ± 1.23
C	55.59 ± 4.64	51.48 ± 6.17	72.04 ± 7.02	5.90 ± 3.13	10.53 ± 1.00
D	58.88 ± 10.01	56.17 ± 9.27	83.21 ± 14.01	4.84 ± 2.29	10.71 ± 0.93
After exercise/before treatment					
A	39.14 ± 7.20	36.83 ± 7.05	51.84 ± 11.64	6.36 ± 1.96	7.38 ± 1.42
B	37.23 ± 5.44	35.53 ± 5.34	49.52 ± 4.58	5.04 ± 1.55	7.11 ± 1.07
C	38.16 ± 3.42	36.48 ± 3.31	52.59 ± 3.40	5.51 ± 2.61	7.30 ± 0.67
D	39.89 ± 6.22	38.20 ± 6.32	56.69 ± 10.13	4.49 ± 3.34	7.67 ± 1.26
After treatment					
A	43.56 ± 7.78	41.18 ± 7.27	57.93 ± 12.15	5.67 ± 2.43	8.23 ± 1.46
B	47.93 ± 6.37 *	45.89 ± 6.31 *	64.11 ± 6.34 *	4.64 ± 2.05	9.18 ± 1.26 *
C	48.57 ± 4.62 **	46.41 ± 3.57 **	67.13 ± 6.32 **	4.39 ± 3.55	9.30 ± 0.71 **
D	49.88 ± 7.79 *	47.59 ± 7.74 *	70.50 ± 11.80 *	5.07 ± 2.26	9.51 ± 1.56 *

*: *p* < 0.05, **: *p* < 0.001.

**Table 4:** The rank of immediate subjective feeling before and after treatment.

	Subjective Feeling					
Before	A Little Tired	Tired	Very Tired	Quite Tired	Burnout	Mean Rank
Group A	1	3	4	1	0	17.33 ± 1.22
Group B	1	4	3	1	0	17.22 ± 1.20
Group C	2	1	4	1	1	17.22 ± 1.86
Group D	1	4	4	0	0	17.11 ± 1.05
After treatment	A little tired	Easy	Easily	Very easily	No sweat at all	Mean rank
Group A	6	3	0	0	0	13.67 ± 0.50 *
Group B	2	5	1	0	1	12.11 ± 2.26 **
Group C	2	3	1	2	1	10.89 ± 2.85 **
Group D	1	6	2	0	0	12.22 ± 1.79 *

**: *p* < 0.05, *: *p* < 0.001. The number N means N people, for example, the number 1 of “Group A; A Little Tired” means there is only one person who felt a little tired in Group A.

**Table 5:** Average peak torque and fatigue level before and after treatment within each group.

	Group A	Group B	Group C	Group D
	Control Group	35 °C	40 °C	45 °C
	Mean ± SD	Mean ± SD	Mean ± SD	Mean ± SD
Average peak torque (Nm)				
Before treatment	36.83 ± 7.05	35.53 ± 5.34	36.48 ± 3.31	38.20 ± 6.32
After treatment	41.18 ± 7.27	45.89 ± 6.31	46.41 ± 3.57	47.59 ± 7.74
MD (95% CI)	−4.34 (−11.50: +2.81)	−10.36 (−16.21: −4.50)	−9.93 (−13.37: −6.49)	−9.39 (−16.47: −2.31)
	*p* = 0.216	*p* = 0.002	*p* = 0.000	*p* = 0.013
Fatigue level				
Before treatment	17.33 ± 1.22	17.22 ± 1.20	17.22 ± 1.86	17.11 ± 1.05
After treatment	13.67 ± 0.50	12.11 ± 2.26	10.89 ± 2.85	12.22 ± 1.79
MD (95% CI)	+3.67 (+2.69: +4.64)	+5.11 (+3.25: +6.97)	+6.33 (+3.90: +8.77)	+4.89 (+ 3.39: +6.38)
	*p* = 0.000	*p* = 0.000	*p* = 0.000	*p* = 0.000

**Table 6:** Comparison of average peak torque and fatigue level after treatment between the experimental group and the control group.

Average Peak Torque (Nm)					
A	41.18 ± 7.27	A	41.18 ± 7.27	A	41.18 ± 7.27
B	45.89 ± 6.31	C	46.41 ± 3.57	D	47.59 ± 7.74
MD (95% CI)	−4.71 (−11.52: +2.09)	MD (95% CI)	−5.23 (−10.96: +0.49)	MD (95% CI)	−6.41 (−13.92: +1.10)
	*p* = 0.162		*p* = 0.071		*p* = 0.089
Fatigue level					
A	13.67 ± 0.50	A	13.67 ± 0.50	A	13.67 ± 0.50
B	12.11 ± 2.26	C	10.89 ± 2.85	D	12.22 ± 1.79
MD (95% CI)	+1.56 (−0.08: +3.19)	MD (95% CI)	+2.78 (+0.73: +4.82)	MD (95% CI)	+1.44 (+0.05: +2.84)
	*p* = 0.061		*p* = 0.011		*p* = 0.044

**Table 7:** Comparison of effects of different fatigue recovery methods.

Study	Participants	Study Design	Mode	Main Results
Kinugasa et al. [48]	28 healthy males	Before the game, after the game, and 24 h after the game	Cold-water immersion	No significant differences were found (*p* > 0.05).
Wang et al. [57]	26 national men’s weightlifting team athletes	Randomized controlled trial	Chinese medicine scraping	The time domain index, frequency domain index, and PSQI in the scraping group were better than those in the control group.
Delextrat et al. [49].	8 men and 8 women basketball players	Randomized controlled trial	Massage, cold-water immersion or control	CWI improves jump performance, also is more useful than massage in recovery, especially in women.
Fiona et al. [50]	34 recreationally active males	Randomized pretest–post-test control	Five post exercise recovery strategies (CWI, CWT, ACT, CONT, COMB).	CWT has better perception recovery, ACT and CONT have better jumping performance than CWI and COMB.
Katsuura et al. [51]	7 healthy male students	Cross-over, control-innovative garments trial	Bioceramic t-shits and trousers	VO_2_ had no significant change (*p* > 0.05).
Nunes et al. [52]	20 elite futsal players	Double-blind placebo-controlled trial	Bioceramic (BIO; *n* = 10); Placebo pants (pl; *n* = 10)	No significant differences were found in 5-metre sprints in week 2.
He et al. [53]	24 athletes with acute injuries	Randomized controlled trial	Massage therapy, ordinary far-infrared therapeutic apparatus, far-infrared ceramic bead therapy	After 3, 7, 14 days of treatment, the indexes of the ceramic bead group were better than those of the other two groups.
This work	36 healthy male students	Randomized controlled trial	Far-infrared graphene heating device	The peak torque and stimulation difference at 40 °C recovery is very significant. The self-perception scale increased significantly after treatment at 35 °C and 40 °C.

## Data Availability

Not applicable.
